# Construction of the Multi-Epitope HFMD Vaccine Based on an Attenuated CVB3 Vector and Evaluation of Immunological Responses in Mice

**DOI:** 10.3390/vaccines14040294

**Published:** 2026-03-26

**Authors:** Jiayi Zheng, Huixiong Deng, Zhuangcong Liu, Hengyao Zhang, Guangzhi Liu, Yanlei Li, Jiacheng Zhu, Liming Gu, Dongdong Qiao, Gefei Wang, Rui Li

**Affiliations:** 1 Department of Microbiology and Immunology, Shantou University Medical College, Shantou 515041, China; 23jyzheng@stu.edu.cn (J.Z.); hxdeng@stu.edu.cn (H.D.); 24zcliu@stu.edu.cn (Z.L.); 22hyzhang@alumni.stu.edu.cn (H.Z.); 24gzliu@stu.edu.cn (G.L.); 22jczhu@alumni.stu.edu.cn (J.Z.); 13lmgu@stu.edu.cn (L.G.); qiaodd5@stu.edu.cn (D.Q.); 2 Department of Obstetrics, The First Affiliated Hospital of Shantou University Medical College, Shantou 515041, China

**Keywords:** hand, foot, and mouth disease, multivalent vaccine, mucosal immunity, immunodominant epitope, vaccine

## Abstract

**Background/Objectives:** Hand, foot, and mouth disease (HFMD) is a major public health concern primarily caused by human enterovirus A71 (EV-A71), coxsackievirus A16 (CVA16), coxsackievirus A6 (CVA6), and certain coxsackievirus B serotypes. Currently available EV-A71 vaccines lack cross-protective efficacy against other serotypes, highlighting the urgent need for multivalent and broadly effective enterovirus vaccines. **Methods:** Immunoinformatics approaches were used to predict highly immunogenic B-cell and T-cell epitopes, which were assembled to construct a novel multivalent epitope vaccine, rCV-A3V, followed by in silico validation. Recombinant protein expression was confirmed by Western blotting and immunofluorescence assays. The immunogenicity was evaluated in Balb/c mice following intranasal immunization. **Results:** A preliminary safety evaluation demonstrated that the rCV-A3V vaccine was well tolerated in the mouse model, with no abnormal changes in body weight observed after immunization. In addition, the target protein was successfully expressed. Intranasal immunization induced a strong Th1-biased immune response, robust serum neutralizing and IgG antibody responses, and pronounced mucosal immunity, including elevated sIgA and IgG levels in nasal lavage fluid, sIgA in feces, and substantial sIgA responses in milk. Dominant epitope peptides were also identified. **Conclusions:** The intranasal live attenuated rCV-A3V vaccine successfully induced humoral, mucosal, and cellular immune responses against EV-A71, CVA16, CVA6, and CVB3, demonstrating broad immunogenicity. These findings provide experimental evidence supporting its potential as a candidate vaccine for HFMD.

## 1. Introduction

Hand, foot, and mouth disease (HFMD) is a highly contagious illness caused by enterovirus infections and predominantly affects children younger than 5 years, although adolescents and adults may also be infected [1]. Over recent decades, HFMD has become a major public health concern in the Asia–Pacific region and worldwide [2]. Enteroviruses are nonenveloped, positive-sense, single-stranded RNA viruses belonging to the *Picornaviridae* family, with a diameter and genome size of approximately 30 nm and 7.4 kb, respectively. To date, more than 250 enterovirus serotypes have been identified, including polioviruses, coxsackievirus group A (CVA, types 1–22 and 24), coxsackievirus group B (CVB, types 1–6), enteroviruses (types 68–71), echoviruses, and rhinoviruses [3,4]. Historically, human enterovirus A71 (EV-A71) and coxsackievirus A16 (CVA16) have been recognized as the principal causative agents of HFMD [5]. However, accumulating epidemiological evidence indicates that coxsackievirus A6 (CVA6) and coxsackievirus A10 (CVA10) have increased markedly in prevalence during HFMD outbreaks worldwide and have become predominant pathogens in certain regions, including parts of China, Vietnam, Finland, Thailand, and France [6,7,8,9]. In addition, coxsackievirus B3 (CVB3), a major etiologic agent of viral myocarditis, poses a growing epidemic risk in China [10]. Although HFMD is generally self-limiting and mild, it may progress to a wide range of clinical outcomes, from uncomplicated disease to severe illness with serious neurological sequelae and, in rare cases, death, particularly among newborns and young children. Typical clinical manifestations include fever accompanied by papulovesicular rashes on the palms, soles, and buttocks, as well as acute flaccid paralysis, pulmonary edema, heart failure, aseptic meningitis, and other uncommon complications [11,12]. Presently, only three formaldehyde-inactivated EV-A71 vaccines have been approved for marketing in China and are manufactured by Beijing Sinovac Biotech Co., Ltd., Wuhan Institute of Biological Products Co., Ltd., and the Institute of Medical Biology, Chinese Academy of Medical Sciences [13,14,15]. Implementation of these vaccines has effectively reduced EV-A71 circulation, resulting in a substantial decline in severe cases and mortality. Nevertheless, monovalent EV-A71 vaccines do not confer cross-protection against other enterovirus serotypes, contributing to pronounced shifts in the epidemiological profile of HFMD in China [12,16]. Consequently, the development of multivalent, highly effective, and broad-spectrum enterovirus vaccines remains an urgent public health priority.

To prevent or control severe sequelae and complications caused by enteroviruses, substantial efforts are underway to advance the development of diverse vaccine platforms. Conventional inactivated vaccines, live attenuated vaccines, and subunit vaccines typically consist of whole pathogens, attenuated vectors, or selected pathogen-derived proteins. These approaches have proven highly effective against infectious agents with limited antigenic variability, such as those causing poliomyelitis, smallpox, rubella, measles, and tetanus [17]. However, their effectiveness is reduced against pathogens characterized by complex immune evasion mechanisms and high mutation rates. Moreover, these vaccines may induce antigenic overload, increasing the risk of adverse reactions, including allergic responses and fever. The presence of nonprotective or weakly immunogenic epitopes may also dilute or interfere with the immunoprotective effects of dominant epitopes. Consequently, there is a clear need to improve vaccine specificity, efficacy, and safety [18,19,20]. Advances in immunoinformatics have transformed traditional vaccine development paradigms and established a novel framework for rational vaccine design. Epitope vaccines represent a class of peptide-based vaccines that use selected immunogenic epitopes derived from pathogens or disease-associated antigens as core components, generated through chemical synthesis or genetic engineering. The underlying principle involves mimicking the key immunologically active regions of native antigens using artificially synthesized epitope peptides. These peptides bind to major histocompatibility complex molecules, thereby activating T-cell–mediated immune responses and stimulating B cells to produce neutralizing antibodies. By rationally integrating B-cell epitopes, cytotoxic T lymphocyte (CTL) epitopes, and helper T lymphocyte epitopes into a single construct, epitope vaccines can simultaneously activate humoral and cellular immunity, resulting in coordinated immune protection and sustained immune memory [21,22,23,24]. A critical determinant of epitope vaccine design is the accurate prediction of highly immunogenic B-cell and T-cell epitopes capable of eliciting targeted immune responses while minimizing reactivity to potentially deleterious epitopes [25]. Immunoinformatics-driven integration of immunogenetics, immunogenomics, and bioinformatics tools enables in silico modeling and evaluation of vaccine constructs, thereby improving development efficiency and facilitating the design of vaccines with minimal adverse effects [26]. Compared with conventional vaccines, epitope vaccines offer several technical advantages. Their short peptide composition reduces the likelihood of allergic or autoimmune reactions, enhancing overall safety. Moreover, they elicit highly specific immune responses, thereby improving immunoprotective efficacy, while also shortening development timelines and reducing production costs [27,28]. Numerous studies have reported the design of epitope-based vaccines targeting *severe acute respiratory syndrome coronavirus 2* (SARS-CoV-2), *Helicobacter pylori*, *Human metapneumovirus*, HFMD-associated pathogens, and other infectious agents [29,30,31,32]. Despite these advances, the immunogenicity of epitope vaccines remains substantially lower than that of whole pathogens or full-length protein antigens, often failing to induce robust and durable immune responses. Accordingly, the use of adjuvants or highly efficient delivery systems is necessary to enhance immunogenicity [33,34]. Enteroviruses are primarily transmitted via the fecal–oral route and respiratory droplets, with the respiratory and gastrointestinal tracts serving as the principal sites of viral replication [3]. Systemic immunity alone often provides suboptimal protection at these mucosal surfaces. Therefore, the central challenge addressed in this study is the development of a vaccine platform capable of delivering multivalent epitopes while effectively inducing both systemic and mucosal immune responses.

The mucosa constitutes the first line of defense against pathogen invasion in the human body. As a major entry route for HFMD viruses, effective activation of local immune responses in the respiratory mucosa is critical. Mucosa-associated lymphoid tissue, the central component of the mucosal immune system, is widely distributed across mucosal sites, including the respiratory tract, gastrointestinal tract, and reproductive system. These tissues include nasopharynx-associated lymphoid tissue (NALT) in the upper respiratory tract, bronchus-associated lymphoid tissue in the lower respiratory tract, gut-associated lymphoid tissue, and other mucosa-associated lymphoid structures [35,36]. Traditional vaccines are predominantly administered by intramuscular injection and primarily induce systemic IgG responses. However, this route is inefficient at activating mucosal immunity and fails to establish an effective first-line defense in the respiratory tract, allowing viruses to enter the host via mucosal surfaces [37]. In contrast, intranasal immunization can concurrently activate mucosal and systemic immune responses, thereby providing dual-layer protection. Intranasal vaccination directly targets the respiratory mucosa, efficiently inducing local immune responses and promoting the production of antigen-specific IgA and resident memory immune cells in the nasal cavity and bronchoalveolar regions, which in turn limit viral replication and transmission. Simultaneously, systemic immune activation leads to the generation of serum IgG antibodies that protect distal target tissues. Compared with intramuscular vaccination, intranasal immunization markedly enhances IgA production and induces IgG antibodies with higher affinity and stronger neutralizing activity [38,39]. By mimicking the natural route of viral infection, this approach promotes durable immune memory while reducing the risk of systemic adverse reactions [40]. As a noninvasive immunization strategy, intranasal vaccination also improves acceptability among young children and is well-suited for large-scale immunization of HFMD high-risk populations [41]. Given the predominant transmission routes of enteroviruses, intranasal immunization represents a more appropriate vaccination strategy. Currently approved mucosal vaccines are mainly live attenuated or inactivated formulations, such as the oral attenuated poliovirus vaccine and the oral inactivated cholera vaccine [42]. To date, the U.S. Food and Drug Administration has approved only nine mucosal vaccines for human use, among which only the live attenuated influenza vaccine FluMist is administered intranasally, whereas the remaining vaccines are delivered orally [35]. Notably, mucosal vaccines have shown considerable potential for preventing a range of viral infections, including influenza virus, SARS-CoV-2, respiratory syncytial virus, and rotavirus, providing valuable insights for the development of next-generation HFMD vaccines [43,44].

Recombinant vector vaccines are capable of inducing immune responses mediated by B cells, CD4+ T cells, and CD8+ T cells, using bacteria or viruses as delivery vectors [45]. Attenuated viral vectors possess several advantageous characteristics for vaccine delivery, including high immunogenicity, the ability to mimic natural infection, and the induction of robust cellular and humoral immune responses, encompassing neutralizing antibodies and T- and B-cell responses. Their favorable safety profile in healthy individuals and relatively low production costs make them an attractive platform for enhancing epitope immunogenicity [46]. The selection of attenuated CVB3 as a vector for HFMD vaccine development offers several distinct advantages. First, CVB3 itself is a clinically relevant HFMD-associated pathogen, allowing the vector to contribute intrinsic antigenic components. Second, CVB3 exhibits natural mucosal tropism, making it particularly suitable for intranasal administration and the activation of mucosal immunity in both the respiratory and intestinal tracts. Third, Deng Huixiong et al. demonstrated through 25 serial passages and RNA sequencing analyses that attenuated CVB3 retains stable attenuation during accelerated viral evolution, supporting its suitability as a reliable vaccine vector. Finally, attenuated CVB3 can function as a gene delivery platform capable of stably expressing exogenous genes, including cytokines or antigenic epitopes, thereby enabling the construction of multivalent epitope vaccines targeting HFMD pathogens [46,47]. Accordingly, the combination of an attenuated CVB3 vector with selected antigenic epitopes, delivered via the intranasal route, is expected to produce a synergistic effect involving the vector, antigen, and delivery pathway. This strategy has the potential to establish an effective immune barrier at viral entry sites while simultaneously activating cellular, humoral, and mucosal immune responses.

Thus, we herein aimed to develop a novel multivalent HFMD vaccine and evaluate its immunogenicity in a mouse model. Candidate epitopes were identified using bioinformatics approaches, and the physicochemical and immunological properties of the designed immunogens were assessed with immunoinformatics tools. Recombinant CVB3 (rCVB3, mu) was used as the vaccine vector. In vitro expression of the immunogen proteins in Vero cells was confirmed by immunofluorescence and Western blotting analyses. Immunogenicity of the multivalent HFMD vaccine was evaluated in Balb/c mice following intranasal immunization. Serum neutralizing antibody titers and IgG binding antibody levels were measured, along with the IgG and sIgA levels in nasal lavage fluid and sIgA levels in fecal samples, to identify dominant epitope peptides. In addition, splenic cellular immune responses were analyzed by flow cytometry.

## 2. Materials and Methods

### 2.1. Prediction and Analysis of Candidate Epitopes

Download the protein sequences of CVA16 (KX595294.1), CVA6 (MF838736.1), and EV-A71 (KP861243.1) from the NCBI database for epitope prediction of candidate vaccines. Linear B-cell epitopes of the viral strains were predicted using the IEDB (https://www.iedb.org/ accessed on 14 November 2022) ABCpred (http://crdd.osdd.net/raghava/abcpred/ accessed on 15 November 2022), and BCPreds (http://ailab.ist.psu.edu/bcpred/ accessed on 15 November 2022) databases. T helper (Th) epitopes were predicted using the IEDB and Rankpep (http://imed.med.ucm.es/Tools/rankpep.html accessed on 16 November 2022) databases. CTL epitopes were predicted using the IEDB, Rankpep, CTLpred (http://crdd.osdd.net/raghava/ctlpred/ accessed on 18 November 2022), and NetCTL server 1.2 (http://www.cbs.dtu.dk/services/NetCTL/ accessed on 19 November 2022). Interferon-gamma (IFN-γ)–inducing and non–interleukin (IL)-4–inducing antigens were predicted using the IFNepitope (https://webs.iiitd.edu.in/raghava/ifnepitope/scan.php accessed on 21 November 2022) and IL4pred (https://webs.iiitd.edu.in/raghava/il4pred/design.php accessed on 22 November 2022) databases. From the predicted epitope pool, epitopes capable of inducing IFN-γ (Th1) but not IL-4 (Th2) responses were selected. Furthermore, previously reported functionally validated effector T-cell epitopes, B-cell neutralizing epitopes, and CTL epitopes were included as references. The ANTIGENpro database (http://scratch.proteomics.ics.uci.edu/ accessed on 23 November 2022) was used to predict the immunogenicity of B-cell and Th epitopes, while the IEDB MHC class I immunogenicity tool was used to assess CTL epitope immunogenicity. Epitope toxicity was evaluated using the protein scanning module of the ToxinPred database (https://webs.iiitd.edu.in/raghava/toxinpred/multi_submit.php accessed on 23 November 2022), and allergenicity was predicted using the AllerTOP v.2.0 database (https://www.ddg-pharmfac.net/AllerTOP/index.html accessed on 24 November 2022). MHC class I and class II binding affinities of candidate CTL and Th epitopes were predicted using the corresponding IEDB tools. For both MHC class I and class II predictions, the default recommended epitope predictor NetMHCpan 4.1 EL was applied. “Human” was selected as the MHC species, along with the corresponding HLA allele reference set. Prediction outputs included half-maximal inhibitory concentrations, percentile ranks, and prediction scores. Peptides with a half-maximal inhibitory concentration of <50 nM were classified as high-affinity binders, with lower percentile ranks indicating stronger binding affinity.

### 2.2. Construction of HFMD Candidate Vaccines

**Figure 1:**
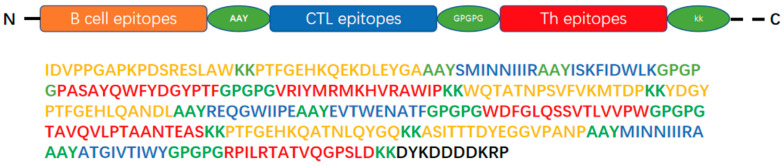
Schematic diagram of the candidate vaccine epitopes.

B-cell epitopes, CTL epitopes, and Th epitopes were linked using AAY, GPGPG, and KK linkers, respectively. Epitopes were sequentially arranged in the order of EV-A71, CVA16, and CVA6. Physicochemical property predictions and immunogenicity assessment results were used to determine the optimal epitope arrangement. A schematic diagram of the epitope vaccine construction is presented in Figure 1.

### 2.3. Physicochemical Properties Analysis of Candidate Vaccines

Comprehensive evaluations of the full-length immunogen sequence were conducted to assess immunogenicity, toxicity, allergenicity, solubility, and physicochemical properties. VaxiJen v2.0 was used to predict the antigenicity of the immunogen. This server employs a machine learning-based algorithm for high-throughput screening of pathogen proteomes to identify potential vaccine candidates. Its main advantage is that it bypasses the need for traditional in vitro experiments, thereby significantly shortening the initial screening stage of vaccine development. In this study, predictions were performed using the viral model, and epitopes with scores > 0.4 were considered candidate epitopes with antigenic potential [48]. The ExPASy ProtParam (https://web.expasy.org/protparam/ accessed on 10 March 2023) server was used to analyze physicochemical characteristics, including molecular weight, theoretical isoelectric point, amino acid composition, atomic composition, estimated half-life, instability index, aliphatic index, and grand average of hydropathicity, among other parameters.

### 2.4. Development of Multivalent HFMD Live-Attenuated Viral Vector Vaccine Using rCVB3 (mu) as the Vector

Using the previously constructed epitope vaccine as the antigenic fragment, a multivalent HFMD vaccine, rCV-A3V, was developed using rCVB3 (mu) as the viral vector. Codon optimization was performed without altering the amino acid sequence, using *Escherichia coli* as the expression host. The epitope antigens were fused and expressed through the viral vector and consisted solely of the antigenic epitope expression sequence, with a FLAG tag added at the 3′ end. The expression gene sequence was made by Guangzhou Aiji Biotechnology Co., Ltd. (Guangzhou, China). The recombinant plasmid pCV-A3V was transformed into *E. coli* Top10 competent cells and plated on LB agar containing kanamycin for single-colony selection and amplification. Positive colonies were selected, and plasmids were extracted and confirmed by sequencing at Guangzhou Aiji Biotechnology Co., Ltd. P0 virus was generated by cotransfecting the correctly sequenced plasmid and pcDNA3.1-T7RNP into 293T cells. The recombinant virus was rescued through blind passage in Vero cells. Viral particles were purified by sucrose gradient ultracentrifugation, aliquoted, and stored at −80 °C.

### 2.5. Protein Detection

The expression of rCV-A3V was analyzed by immunofluorescence staining and Western blotting according to the procedures described in the Supplementary Materials.

### 2.6. Immunization of Mice

SPF-grade 8-week-old Balb/c mice were used for immunization studies with the rCV-A3V vaccine. Mice were purchased from Guangdong Vital River Laboratory Animal Technology Co., Ltd. (Foshan, Guangdong, China). All experimental procedures were conducted in accordance with the National Institutes of Health Guide for the Care and Use of Laboratory Animals and were approved by the Animal Care and Use Research Ethics Committee of Shantou University Medical College (Approval No.: SUMC-2021-17). Balb/c mice were randomly assigned to the control group, rCVB3 (mu) group, or rCV-A3V vaccine group (*n* = 10 per group). During immunogen preparation, differences were observed in the virus purification and concentration processes between rCVB3 (mu) and rCV-A3V. Following the median tissue culture infectious dose 50 (TCID50) assay, the final immunization doses were determined as follows: the rCVB3 (mu) group received 50 μL of 7 × 10^4^ TCID50/mL CVB3 (mu) via intranasal instillation; the rCV-A3V vaccine group received 50 μL of 10^3^ TCID50/mL rCV-A3V; and the control group received an equal volume of PBS. A three-dose immunization regimen was applied on days 0, 14, and 28. Fourteen days after the second immunization, six mice from each group were bled via the facial vein plexus. Fourteen days after the third immunization, six mice from each group were bled via the retro-orbital venous plexus, and sera were isolated for antibody detection. In addition, six mice from each group were euthanized 14 days after the third immunization, and their spleens were collected for T-cell responsiveness analysis. Concurrently, nasal lavage fluid and fecal samples were collected for mucosal antibody detection. The remaining male and female mice were co-housed after the third immunization and produced litters approximately 20 days later. Milk spots were collected from the suckling pups 6 days after birth to assess sIgA antibody levels. On day 6 postpartum, 2–3 suckling mice were randomly selected from each litter, and milk spots were collected individually from each pup as independent samples. A mixed-effects model was used to account for within-litter correlation. Because some female mice failed to become pregnant, milk spot samples were ultimately collected from a total of 5 suckling mice per group. Cages and water bottles were replaced every 2 days until the end of the experiment. Throughout the study, mice had free access to food and water, were monitored twice daily, and body weight was recorded every 2 days.

### 2.7. Neutralizing Antibody Detection

Vero cells and RD cells were obtained from the Cell Bank of the Chinese Academy of Sciences (Shanghai, China). The strains EV-A71, CVA16, CVA6 (M11), and CVB3 (Nancy) were preserved in our laboratory. Vero cells and RD cells were seeded into 96-well plates at a density of 0.25 × 10^6^ cells/mL. When cell confluence reached 90–100%, viral infection assays were performed. EV-A71, CVA16, and CVB3 were used to infect Vero cells, whereas CVA6 was used to infect RD cells. Prior to testing, serum samples were heat-inactivated at 56 °C for 30 min and serially diluted twofold from 1:10 to 1:320. Equal volumes of virus suspension containing 100 TCID_50_/mL and diluted serum were mixed and incubated at 37 °C in a 5% CO_2_ incubator for 1 h. Following incubation, 100 μL/well of each mixture was added to the corresponding 96-well plates and incubated for 1 h. Subsequently, complete DMEM (Gibco, 8122622, Newark, DE, USA) supplemented with 10% fetal bovine serum (Lonsera, PF09666, Ciudad de la Costa, Uruguay), and 100 U/mL penicillin and 100 μg/mL streptomycin (Gibco, 15140-122) was added at 100 µL/well concentration into a 96-well plate, with three replicate wells established for each serum dilution. A virus control and a cell control were simultaneously set up. The cytopathic effects were monitored within 3 days. The highest serum dilution that completely protected cells from cytopathic effects was defined as the neutralizing antibody titer.

### 2.8. Enzyme-Linked Immunosorbent Assay (ELISA) Analysis

Purified viral solutions of EV-A71, CVA16, CVA6, and CVB3 were inactivated, and protein concentrations were determined using a BCA protein assay kit (Beyotime, P0010, Shanghai, China). The solutions were diluted to 1 μg/mL in 1× coating buffer (0.05 mol/L Na_2_CO_3_-NaHCO_3_ buffer, pH 9.6) and added to 96-well plates at 100 μL/well, followed by overnight incubation at 4 °C. Plates were washed five times with 1× PBST (1× PBS/0.1% Tween-20) and blocked with blocking buffer (1× PBST/1% BSA, pH 7.4) at 37 °C for 1 h prior to sample addition. Serum samples were subjected to fivefold serial dilutions from 1:125 to 1:15,625, whereas nasal lavage fluid, fecal extracts, and milk samples were diluted fivefold from 1:5 to 1:625, with two replicate wells per dilution. Plates were incubated at 37 °C for 1–1.5 h and then washed. Horseradish peroxidase (HRP)-conjugated goat antimouse IgG (Beyotime, A0216) or HRP-conjugated goat antimouse IgA (Abcam, ab97235, Cambridge, UK) was added as the secondary antibody and incubated at 37 °C for 1 h. Subsequently, 100 μL/well of TMB substrate solution (Beyotime, P0209-500 mL) was added for color development. The reaction was terminated by adding 100 μL/well of sulfuric acid–free TMB stop solution (Beyotime, P0215-500 mL). Optical density was measured at 450 nm within 10 min using a microplate reader.

### 2.9. Splenocyte Isolation and Flow Cytometry Analysis

After immunization, spleens from Balb/c mice were harvested and suspended in cold PBS. Tissues were mechanically homogenized using a grinding rod and sequentially filtered through 100 μm and 70 μm cell strainers (BD Biosciences, San Jose, CA, USA). Lymphocyte separation medium was added, followed by centrifugation at 450× *g* for 30 min. The lymphocyte layer was collected, resuspended in PBS, and re-centrifuged at 350× *g* for 10 min. Isolated lymphocytes were cryopreserved in serum-free cell freezing medium for subsequent analyses. Lymphocytes were diluted to 5 × 10^6^ cells/mL in RPMI-1640 medium (Absin, abs9484, Shanghai, China) supplemented with 10% fetal bovine serum (Lonsera, OC08226), 100 U/mL penicillin, and 100 ug/mL streptomycin (Gibco, 15140-122), and incubated at 37 °C in a 5% CO_2_ incubator for 6 h. The positive stimulation group was treated with a positive stimulant (Invitrogen, 00-4970-03, Carlsbad, CA, USA), whereas the PBS control group, rCVB3 group, and rCV-A3V group were incubated with a peptide library mixture at 250 nmol/peptide for 16 h. Cells were washed and blocked using stain buffer (BD Pharmingen, 554656, San Jose, CA, USA) and Fc blocker (Invitrogen, 2866269), followed by surface staining with BV510 anti-CD3e, FITC anti-CD4, and APC anti-CD8a antibodies (BD Pharmingen). Cells were then fixed and permeabilized using a fixation/permeabilization kit (BD Pharmingen), followed by intracellular staining with PE rat antimouse IFN-γ and PE-Cy7 rat antimouse IL4 antibodies (BD Pharmingen). Data acquisition and analysis of 10,000 events per sample were performed using a CYTEK Aurora flow cytometer and FlowJo_V10 software.

### 2.10. Statistical Analysis

Statistical analyses were performed using GraphPad Prism 10.1.2. One-way ANOVA, two-way ANOVA, and Tukey’s multiple comparison test were applied as appropriate. For flow cytometry data analysis, Grubbs’ test (α = 0.05) was used to identify outliers for each measured parameter. Samples identified as outliers were excluded from the analysis of the respective parameter to minimize the impact of technical artifacts on the statistical results. The sample sizes indicated in the figures represent the effective sample sizes after outlier exclusion. Data are presented as the mean ±SEM.

## 3. Results

### 3.1. Prediction and Screening of Antigenic Epitopes

Using immunoinformatics-based prediction and analysis of antigenic epitopes derived from enterovirus EV-A71, CVA16, and CVA6 proteins, candidate B-cell, CTL, and Th epitopes with high immunogenicity, strong sequence conservation, nontoxicity, and nonallergenicity were identified. The selected Th epitopes were characterized by their ability to induce IFN-γ responses. Detailed information on the screened B-cell, CTL, and Th epitopes and their corresponding evaluation parameters is provided in Table 1, respectively.

### 3.2. Design of Multivalent HFMD Vaccine

A multivalent HFMD vaccine, designated rCV-A3V, was designed using rCVB3 (mu) as the viral vector and incorporating B-cell, CTL, and Th epitopes derived from EV-A71, CVA16, and CVA6 (Figure 1). These epitopes were connected using AAY, GPGPG, and KK linkers to preserve their conformational independence and structural flexibility. A FLAG tag (DYKDDDDKRP) was appended to the C-terminus of the immunogen to facilitate protein detection.

### 3.3. Physicochemical Properties of the Candidate Vaccine rCV-A3V

The candidate multivalent epitope vaccine molecule exhibited high immunogenicity, with an overall antigenicity score of 0.4635 predicted by VaxiJen v2.0, exceeding the default threshold for the viral model. It was also predicted to be non-allergenic and non-toxic. The constructed vaccine protein has a molecular weight of 32.1414 kDa and consists of 292 amino acids, with a theoretical isoelectric point of 8.93 and an aliphatic index of 64.59, indicating a hydrophobic core characteristic of globular proteins. The grand average of hydropathicity value was −0.523, suggesting an overall hydrophilic nature. An instability index of 36.8 indicates that the protein is stable. Collectively, these physicochemical characteristics suggest that rCV-A3V is a soluble globular protein with a hydrophilic surface and hydrophobic interior and exhibits good structural stability. The estimated half-life of rCV-A3V is 20 h in mammalian reticulocytes, 30 min in yeast cells, and more than 10 h in *E. coli* (Table 2).

### 3.4. Protein Expression Study of rCV-A3V Vaccine

**Figure 2:**
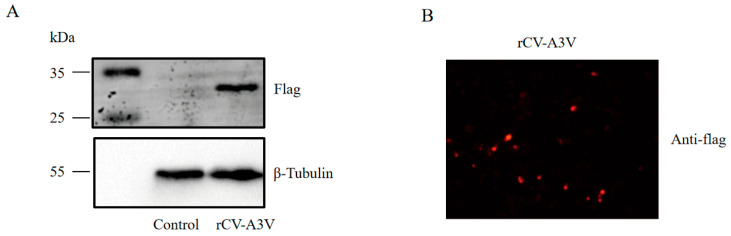
Expression profile of rCV-A3V. (**A**) Detection of rCV-A3V expression by Western blotting. (**B**) Immunofluorescence staining of rCV-A3V expression in Vero cells. Red fluorescence indicates rCV-A3V protein expression.

To assess rCV-A3V protein expression, Vero cells were infected with rCV-A3V P1, and protein expression was detected using an anti-FLAG antibody. Western blotting analysis identified a distinct protein band at the expected molecular weight of 32.1 kDa (Figure 2A). Consistently, immunofluorescence staining revealed strong red fluorescence in rCV-A3V–infected cells (Figure 2B). These findings confirm that the rCV-A3V vaccine efficiently mediates transgene expression and robust protein production in cultured cells.

### 3.5. Immunization and Antibody Response in Mice

**Figure 3:**
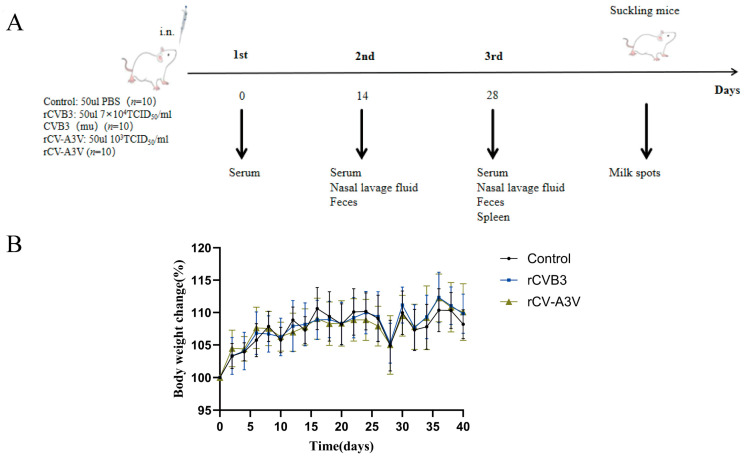

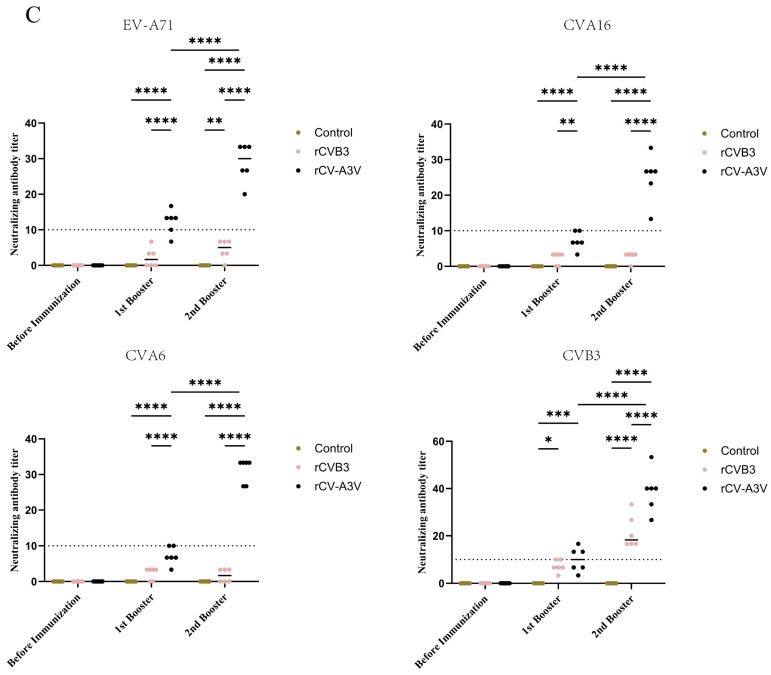
Immunization Schedule and Neutralizing Antibody Response of the rCV-A3V Mucosal Vaccine. (**A**) Immunization schedule. Mice were immunized on days 0, 14, and 28. Preimmune serum was collected before the first immunization. Serum, nasal lavage fluid, and fecal samples were collected 2 weeks after the second and third immunizations. Spleens were also harvested 2 weeks after the third immunization for immune parameter assessment. (**B**) Body weight changes in mice after immunization. (**C**) Detection of virus-neutralizing antibodies (*n* = 6). Using one-way ANOVA and Tukey’s multiple comparison test, EV-A71: ** *p* = 0.0097, **** *p* < 0.0001. CVA16: ** *p* = 0.0028, **** *p* < 0.0001. CVA6: **** *p* < 0.0001. CVB3: * *p* = 0.0128, *** *p* = 0.0005, **** *p* < 0.0001.

**Figure 4:**
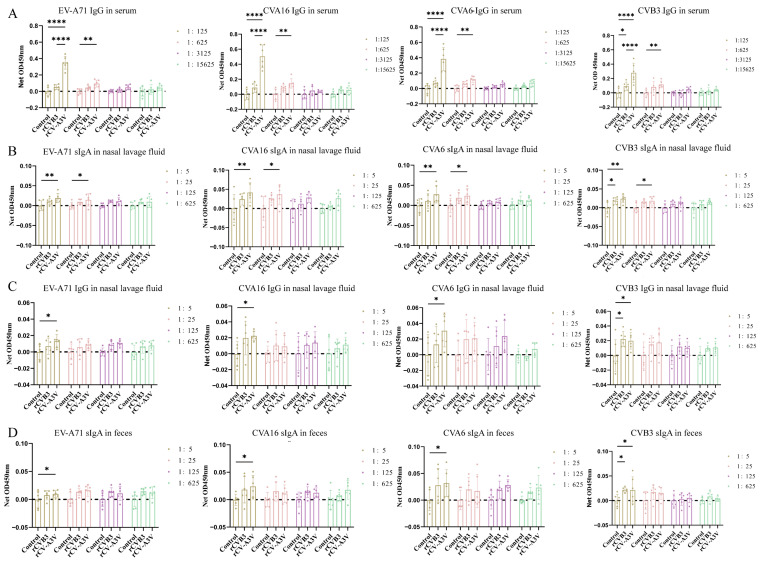
Detection of Antibody Responses After the First Booster Immunization in Mouse Models by Enzyme-linked Immunosorbent Assay (ELISA). (**A**) Antigen-specific immunoglobulin G (IgG) against human enterovirus A71 (EV-A71), coxsackievirus A16 (CVA16), coxsackievirus A6 (CVA6), and coxsackievirus B3 (CVB3) in serum (*n* = 6). (**B**,**C**) Virus-specific secretory immunoglobulin A (sIgA) and IgG against EV-A71, CVA16, CVA6, and CVB3 in nasal lavage fluid (*n* = 6). (**D**) Virus-specific sIgA against EV-A71, CVA16, CVA6, and CVB3 in feces (*n* = 6). Data are presented as net OD450 nm (sample raw OD − mean control OD at same dilution). Dashed line: control baseline. Using one-way ANOVA and Tukey’s multiple comparison test. (**A**): EV-A71: ** *p* = 0.0012, **** *p* < 0.0001. CVA16: ** *p* = 0.0016, **** *p* < 0.0001. CVA6: ** *p* = 0.0013, **** *p* < 0.0001. CVB3: * *p* = 0.0264, ** *p* = 0.0045, **** *p* < 0.0001. (**B**): EV-A71: * *p* = 0.0292, ** *p* = 0.0028. CVA16: * *p* = 0.0137, ** *p* = 0.0046. CVA6: * *p* = 0.0122, ** *p* = 0.0031. CVB3:1:5: * *p* = 0.0140, ** *p* = 0.0015, 1:25: * *p* = 0.0172. (**C**): EV-A71: * *p* = 0.0107. CVA16: * *p* = 0.0357. CVA6: * *p* = 0.0221. CVB3: * *p* = 0.0121 (Control vs. rCVB3), * *p* = 0.0271 (Control vs. rCV-A3V). (**D**): EV-A71: * *p* = 0.0130. CVA16: * *p* = 0.0207. CVA6: * *p* = 0.0249. CVB3: * *p* = 0.0190 (Control vs. rCVB3), * *p* = 0.0224 (Control vs. rCV-A3V).

**Figure 5:**
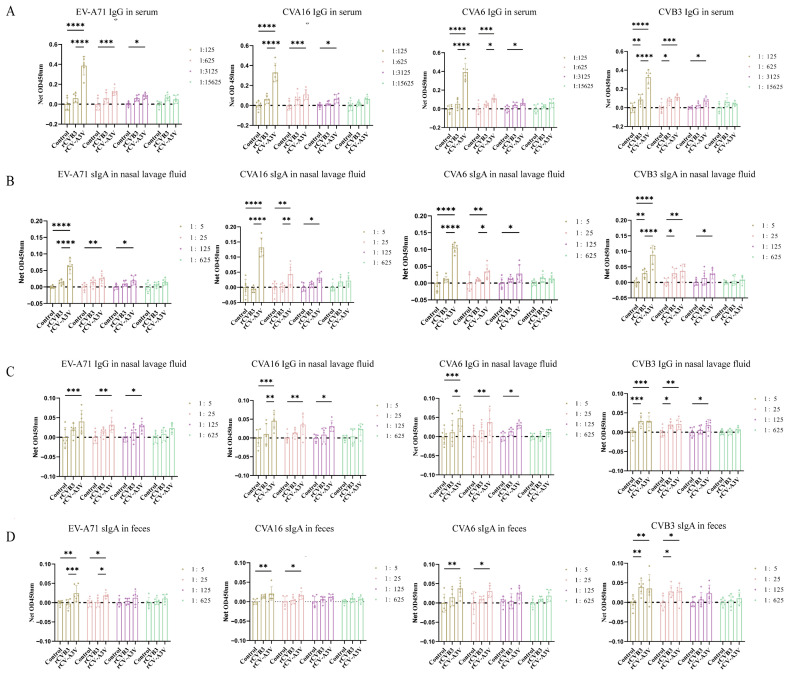
Detection of Antibody Responses After the Second Booster Immunization in Mouse Models by Enzyme-linked Immunosorbent Assay (ELISA). (**A**) Antigen-specific immunoglobulin G (IgG) against human enterovirus A71 (EV-A71), coxsackievirus A16 (CVA16), coxsackievirus A6 (CVA6), and coxsackievirus B3 (CVB3) in serum (*n* = 6). (**B**,**C**) Virus-specific secretory immunoglobulin A (sIgA) and IgG against EV-A71, CVA16, CVA6, and CVB3 in nasal lavage fluid (*n* = 6). (**D**) Virus-specific sIgA against EV-A71, CVA16, CVA6, and CVB3 in feces (*n* = 6). Data are presented as net OD450 nm (sample raw OD − mean control OD at same dilution). Dashed line: control baseline. Using one-way ANOVA and Tukey’s multiple comparison test. (**A**): EV-A71: * *p* = 0.0145, *** *p* = 0.0001, **** *p* < 0.0001. CVA16: * *p* = 0.0481, *** *p* = 0.0006, **** *p* < 0.0001. CVA6: * *p* = 0.0495 (1:625), * *p* = 0.0466 (1:3125), *** *p* = 0.0003, **** *p* < 0.0001. CVB3: * *p* = 0.0114 (1:625), * *p* = 0.0139 (1:3125), ** *p* = 0.0030, *** *p* = 0.0001, **** *p* < 0.0001. (**B**): EV-A71: * *p* = 0.0198, ** *p* = 0.0016, **** *p* < 0.0001. CVA16: * *p* = 0.0223, ** *p* = 0.0012 (Control vs. rCV-A3V), ** *p* = 0.0018 (rCVB3 vs. rCV-A3V), **** *p* < 0.0001. CVA6: * *p* = 0.0310 (1:25), * *p* = 0.0136 (1:125), ** *p* = 0.0018, **** *p* < 0.0001. CVB3: * *p* = 0.0117 (1:25), * *p* = 0.0185 (1:125), ** *p* = 0.0097 (1:5), ** *p* = 0.0013 (1:25), **** *p* < 0.0001. (**C**): EV-A71: * *p* = 0.0209, ** *p* = 0.0075, *** *p* = 0.0006. CVA16: * *p* = 0.0138, ** *p* = 0.0048 (1:5), ** *p* = 0.0044 (1:25), *** *p* = 0.0002. CVA6: * *p* = 0.0103 (1:5), * *p* = 0.0419 (1:125), ** *p* = 0.0078, *** *p* = 0.0006. CVB3: * *p* = 0.0112 (1:25), * *p* = 0.0206 (1:125), ** *p* = 0.0059, *** *p* = 0.0002 (Control vs. rCVB3), *** *p* = 0.0003 (Control vs. rCV-A3V) (**D**): EV-A71: * *p* = 0.0256 (Control vs. rCV-A3V), * *p* = 0.0436 (rCVB3 vs. rCV-A3V), ** *p* = 0.0026, *** *p* = 0.0008. CVA16: * *p* = 0.0247, ** *p* = 0.0016. CVA6: * *p* = 0.0136, ** *p* = 0.0015. CVB3: * *p* = 0.0377 (Control vs. rCVB3), * *p* = 0.0406 (Control vs. rCV-A3V), ** *p* = 0.0018 (Control vs. rCVB3), ** *p* = 0.0005 (Control vs. rCV-A3V).

**Figure 6:**
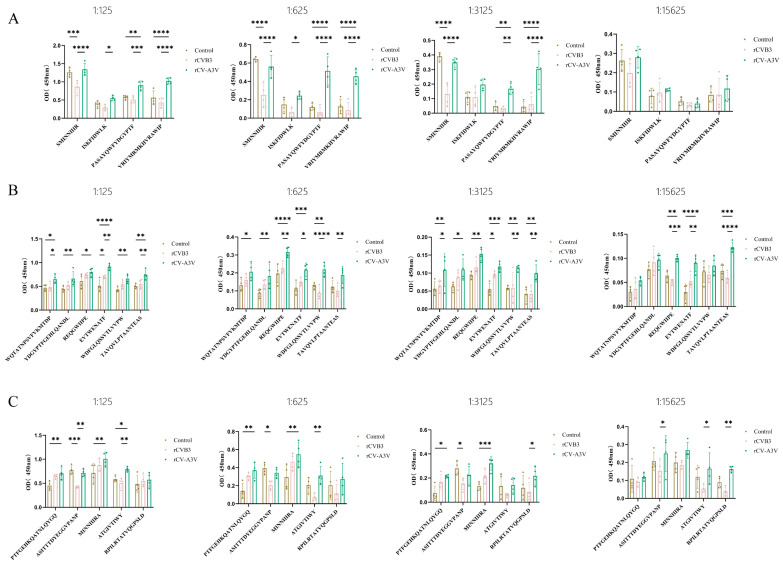
Screening of Immunodominant Epitope Peptides by Enzyme-linked Immunosorbent Assay (ELISA). (**A**) Immunoglobulin G (IgG) against human enterovirus A71 (EV-A71) epitopes in serum (*n* = 4). (**B**) IgG against coxsackievirus A16 (CVA16) epitopes in serum (*n* = 4). (**C**) IgG against coxsackievirus A6 (CVA6) epitopes in serum (*n* = 4). “Dominant epitopes” were defined as those capable of inducing significantly higher specific antibody responses in immunized mice when compared to the negative control, considering the magnitude of the statistical significance. Initially, six mice were included in each group for serum collection. During sample processing, some samples were excluded from subsequent antibody detection owing to insufficient blood collection volume. The final number of samples included in the analysis was 4 per group. Using two-way ANOVA and Tukey’s multiple comparison test. (**A**): 1:125: * *p* = 0.0137, ** *p* = 0.0016, *** *p* = 0.0002, **** < 0.0001. 1:625: * *p* = 0.0132, **** < 0.0001. 1:3125: ** *p* = 0.0076 (Control vs. rCV-A3V), ** *p* = 0.0019 (rCVB3 vs. rCV-A3V), **** < 0.0001. (**B**): 1:125: * *p* = 0.0152 (WQTATNPSVFVKMTDP, Control vs. rCV-A3V), * *p* = 0.0272 (WQTATNPSVFVKMTDP, rCVB3 vs. rCV-A3V), * *p* = 0.0114 (REQGWIIPE, Control vs. rCV-A3V), * *p* = 0.0143 (EVTWENATF, Control vs. rCVB3), ** *p* = 0.0031 (YDGYPTFGEHLQANDL, Control vs. rCV-A3V), ** *p* = 0.0032 (EVTWENATF, rCVB3 vs. rCV-A3V), ** *p* = 0.0017 (WDFGLQSSVTLVVPW, Control vs. rCV-A3V), ** *p* = 0.0010 (TAVQVLPTAANTEAS, Control vs. rCV-A3V), ** *p* = 0.0087 (TAVQVLPTAANTEAS, rCVB3 vs. rCV-A3V), **** < 0.0001. 1:625: * *p* = 0.0175 (WQTATNPSVFVKMTDP, Control vs. rCV-A3V), * *p* = 0.0314 (EVTWENATF, rCVB3 vs. rCV-A3V), * *p* = 0.0443 (TAVQVLPTAANTEAS, Control vs. rCV-A3V), ** *p* = 0.0025 (YDGYPTFGEHLQANDL, Control vs. rCV-A3V), ** *p* = 0.0031 (REQGWIIPE, rCVB3 vs. rCV-A3V), ** *p* = 0.0031 (WDFGLQSSVTLVVPW, rCVB3 vs. rCV-A3V), ** *p* = 0.0040 (TAVQVLPTAANTEAS, rCVB3 vs. rCV-A3V), *** *p* = 0.0006, **** < 0.0001. 1:3125: * *p* = 0.0241 (WQTATNPSVFVKMTDP, rCVB3 vs. rCV-A3V), * *p* = 0.013 (YDGYPTFGEHLQANDL, Control vs. rCV-A3V), * *p* = 0.0306 (EVTWENATF, Control vs. rCVB3), ** *p* = 0.0052 (WQTATNPSVFVKMTDP, Control vs. rCV-A3V), ** *p* = 0.0011 (REQGWIIPE, Control vs. rCV-A3V), ** *p* = 0.0030 (WDFGLQSSVTLVVPW), ** *p* = 0.0018 (TAVQVLPTAANTEAS, Control vs. rCV-A3V), ** *p* = 0.0016 (TAVQVLPTAANTEAS, rCVB3 vs. rCV-A3V), *** *p* = 0.0006, 1:15,625: ** *p* = 0.0049 (REQGWIIPE), ** *p* = 0.0035 (EVTWENATF), *** *p* = 0.0001 (REQGWIIPE, rCVB3 vs. rCV-A3V), *** *p* = 0.0002 (TAVQVLPTAANTEAS, Control vs. rCV-A3V), **** < 0.0001. (**C**): 1:125: * *p* = 0.0394, **: 0.0024 (ASITTTDYEGGVPANP, rCVB3 vs. rCV-A3V), ** *p* = 0.0022 (MINNIIIRA, Control vs. rCV-A3V), ** *p* = 0.0033 (ATGIVTIWY, rCVB3 vs. rCV-A3V), *** *p* = 0.0003, 1:625: * *p* = 0.0292, ** *p* = 0.0072 (PTFGEHKQATNLQYGQ, Control vs. rCV-A3V), ** *p* = 0.0027 (MINNIIIRA, Control vs. rCV-A3V), ** *p* = 0.0046 (ATGIVTIWY, rCVB3 vs. rCV-A3V), 1:3125: * *p* = 0.0110 (PTFGEHKQATNLQYGQ, Control vs. rCV-A3V), * *p* = 0.0211 (ASITTTDYEGGVPANP, Control vs. rCVB3), * *p* = 0.0162 (RPILRTATVQGPSLD, rCVB3 vs. rCV-A3V), *** *p* = 0.0004, 1:15,625: * *p* = 0.0323 (ASITTTDYEGGVPANP, rCVB3 vs. rCV-A3V), * *p* = 0.0121 (ATGIVTIWY, rCVB3 vs. rCV-A3V), ** *p* = 0.0048.

**Figure 7:**
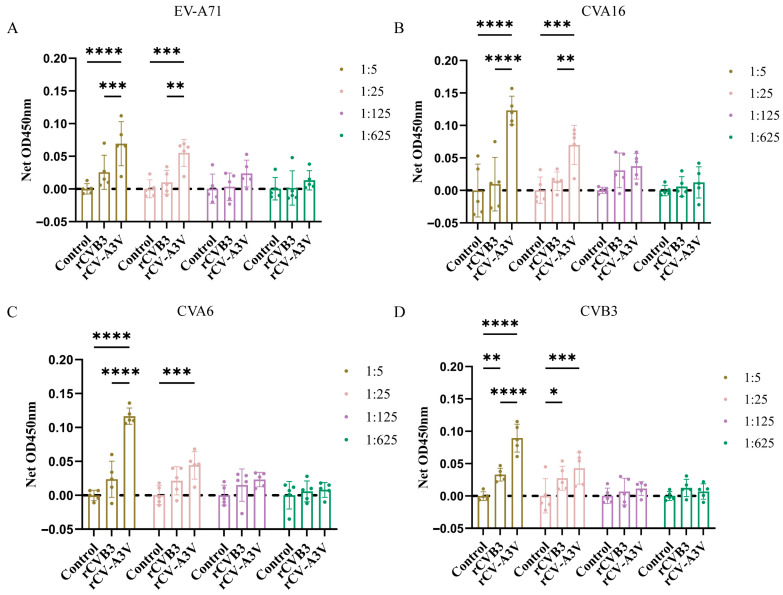
Secretory immunoglobulin A (sIgA) Antibodies in Milk from Suckling Mice Vaccinated with rCV-A3V. (**A**–**D**) Detection of sIgA in milk spots from suckling mice. On day 6 postpartum, 2–3 suckling mice were randomly selected from each litter for milk spot collection. Each data point represents an individual sample from a suckling mouse, with 5 mice per group. Statistical analysis was performed using a mixed-effects model to account for within-litter correlation. Data are presented as net OD450 nm (sample raw OD − mean control OD at same dilution). Dashed line: control baseline. Using one-way ANOVA and Tukey’s multiple comparison test. EV-A71: ** *p* = 0.0020, *** *p* = 0.0001 (1:5), *** *p* = 0.000 (1:25), **** *p* < 0.0001. CVA16: ** *p* = 0.0024, *** *p* = 0.0001, **** *p* < 0.0001. CVA6: *** *p* = 0.0006, **** *p* < 0.0001. CVB3: * *p* = 0.0335, ** *p* = 0.0076, *** *p* = 0.0005, **** *p* < 0.0001.

To evaluate humoral and mucosal immune responses induced by intranasal rCV-A3V immunization, Balb/c mice were selected as the immunization model (Figure 3A). A prime–boost immunization strategy consisting of three intranasal administrations was used to assess serum neutralizing antibody responses elicited by rCV-A3V. We analyzed neutralizing antibody titers against EV-A71, CVA16, CVA6, and CVB3. Two weeks after the second booster immunization, neutralizing antibody titers against all four viruses in sera from immunized mice were significantly higher than those in the control group. Throughout the experimental period, body weights of mice in the rCVB3 and rCV-A3V groups increased steadily and were comparable to those of the control group, indicating good tolerability of the immunization regimen (Figure 3B). Moreover, neutralizing antibody levels increased progressively with successive immunizations (Figure 3C). To further characterize virus-specific antibody responses, levels of immunoglobulin G (IgG) and secretory immunoglobulin A (sIgA) were evaluated. Serum samples were analyzed for virus-specific IgG antibodies against EV-A71, CVA16, CVA6, and CVB3, whereas nasal lavage fluid was assessed for virus-specific IgG and sIgA antibodies, and fecal samples were examined for virus-specific sIgA. Compared with the control group, intranasally immunized mice produced detectable virus-specific IgG antibodies against all four viruses 2 weeks after the first booster immunization. Serum IgG levels against EV-A71, CVA16, CVA6, and CVB3 were significantly higher in immunized mice than in control mice at dilutions of 1:125 and 1:625 (Figure 4A). Consistent with these findings, virus-specific IgG and sIgA antibodies were detected in nasal lavage fluid. Two weeks after the first booster immunization, intranasally immunized mice exhibited significantly higher levels of both IgG and sIgA antibodies against all four viruses compared with control mice (Figure 4B,C). Fecal sIgA levels were also assessed, and 2 weeks after the first booster immunization, intranasally immunized mice showed significantly higher virus-specific sIgA levels at a dilution of 1:5 than those observed in the control group (Figure 4D). Following the second booster immunization, the rCV-A3V group showed markedly higher serum levels of virus-specific IgG antibodies against EV-A71, CVA16, CVA6, and CVB3 than the control group (Figure 5A). Similarly, levels of virus-specific IgG and sIgA in nasal lavage fluid and virus-specific sIgA in feces were further increased relative to both the control group and the levels observed after the first booster immunization (Figure 5B–D). To identify dominant epitope peptides, EV-A71, CVA16, and CVA6 epitope peptides were used as coating antigens in ELISA assays to determine whether epitope-specific IgG antibodies were induced in the sera of immunized mice (Figure 6A–C). Specific IgG antibodies against 15 epitope peptides were detected in sera from the rCV-A3V-immunized group. Among these, the dominant epitope peptide for EV-A71 was VRIYMRMKHVRAWIP, for CVA16 was EVTWENATF, and for CVA6 was MINNIIIRA. To evaluate the potential of maternal–neonatal protection mediated by passive immunity following intranasal rCV-A3V immunization, sIgA antibody levels were measured in the milk of immunized female mice. High titers of virus-specific sIgA antibodies against EV-A71, CVA16, CVA6, and CVB3 were detected in milk samples from the rCV-A3V-immunized group (Figure 7).

### 3.6. Cellular Immune Response Induced by the rCV-A3V Vaccine in Immunized Mice

**Figure 8:**
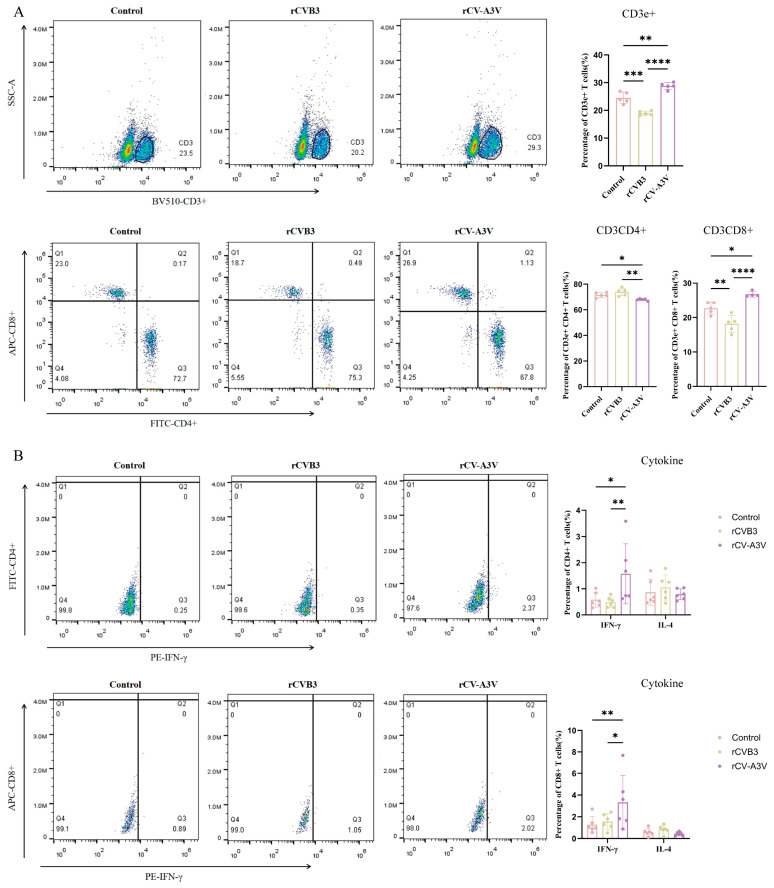
Cellular Immune Response Induced by rCV-A3V. (**A**) Proportions of CD3e^+^, CD4^+^, and CD8^+^ T lymphocytes (*n* = 4–5). (**B**) Interferon-gamma (IFN-γ) secretion by CD4^+^ and CD8^+^ T lymphocytes. Initially, six mice from each group were included for splenic T-cell analysis. During flow cytometry data analysis, Grubbs’ test (α = 0.05) was used to identify outliers, and those identified were excluded from the analysis of the specific parameter. Using one-way ANOVA and Tukey’s multiple comparison test. For CD3+ and CD4+ T cells, *n* = 5 per group; for CD8+ T cells, *n* = 5 in the PBS and rCVB3 (mu) groups and *n* = 4 in the rCV-A3V group; and for IFN-γ+ cells, *n* = 6 per group. CD3e+: ** *p* = 0.0013, *** *p* = 0.0001, **** *p* < 0.0001. CD3CD4+: * *p* = 0.0393, ** *p* = 0.0018. CD3CD8+: * *p* = 0.0178, ** *p* = 0.0066, **** *p* < 0.0001. Using two-way ANOVA and Tukey’s multiple comparison test. IFN-γ(CD3CD4+): * *p* = 0.0146, ** *p* = 0.0072. IFN-γ(CD3CD8+): * *p* = 0.0284, ** *p* = 0.0095.

To evaluate the immunostimulatory effects of the rCV-A3V mucosal vaccine on T-cell responses, lymphocytes were isolated from mouse spleens 2 weeks after the third intranasal immunization. The proportion of CD3e^+^ T cells in the rCV-A3V–immunized group was significantly higher than that in the control group, indicating enhanced overall T-cell activation or expansion. Compared with the control group, the rCV-A3V group exhibited a significantly higher proportion of CD8^+^ T cells and a reduced proportion of CD4^+^ T cells (Figure 8A). Despite this shift, IFN-γ secretion was significantly increased in the rCV-A3V group (Figure 8B), indicating robust functional activation of T cells. These findings suggest that intranasal immunization with rCV-A3V preferentially promotes CD8^+^ T-cell–mediated responses while inducing a pronounced Th1-biased cellular immune profile, as reflected by elevated IFN-γ expression.

## 4. Discussion

The outbreak of enteroviruses has emerged as a global public health concern, particularly in the Asia–Pacific region. Vaccination remains the most effective strategy for preventing the spread of infectious diseases. Although considerable efforts have been made in the development of vaccines for hand, foot, and mouth disease (HFMD), specific therapeutic drugs and effective vaccines remain lacking, underscoring an urgent need for safe and efficacious HFMD vaccines. Multi-epitope synthetic peptide vaccines represent a promising approach to address the co-circulation of multiple enteroviruses. In this study, we successfully constructed and evaluated a novel multivalent intranasal live attenuated vaccine, rCV-A3V. This vaccine integrates antigenic epitopes derived from EVA71, CVA16, and CVA6 with an attenuated CVB3 vector, which was administered intranasally to Balb/c mice. Our findings demonstrated that rCV-A3V elicits robust and comprehensive humoral, cellular, and mucosal immune responses, offering new insights into the development of multivalent broad-spectrum HFMD vaccines and the optimization of immunization strategies.

In this study, a preliminary safety evaluation of the candidate rCV-A3V vaccine was conducted. By continuously monitoring changes in the body weight in mice post-immunization, we noted no significant differences in the body weight gain trends between all immunized groups and the control group, indicating favorable short-term tolerability of the vaccine at the whole-animal level. Notably, the genetic stability of the attenuated CVB3 (mu) vector used in this study was evaluated through rapid evolution cell models and RNA sequencing, and its temperature stability and attenuation characteristics were validated through both in vivo and in vitro experiments in our previous work. After serial passaging in Vero cells up to the 25th passage, rCVB3 (mu) retained its attenuated phenotype and exhibited significantly restricted replication capacity at 37 °C and above. In mice, it displayed favorable attenuation characteristics, with no evident histopathological lesions observed in cardiac or neural tissues [47]. This information provided contextual support for the overall safety design of rCV-A3V. Nevertheless, the current safety data are primarily limited to body weight observations, which represent only one aspect of safety evaluation. To fully confirm the safety of rCV-A3V, future studies should incorporate more detailed experimental data, such as histopathological analysis of major organs and the detection of serum biochemical parameters, to eliminate potential organ-specific toxicity or inflammatory damage.

rCV-A3V induced broad and potent humoral immune responses. Following three immunizations, high levels of IgG and neutralizing antibodies against EVA71, CVA16, CVA6, and CVB3 were detected in mouse sera, with the antibody levels significantly increasing with each subsequent immunization. Notably, the CVB3-neutralizing antibody titers induced by the rCV-A3V group were higher than those induced by the rCVB3 group, suggesting that the integrated EVA71, CVA16, and CVA6 epitopes enhanced the immunogenicity of the vector itself through cross-reactive immune stimulation. Past studies have indicated that incorporating key neutralizing epitopes from the enterovirus VP1 protein into the rCVB3 vector may present a broader array of antigenic determinants, thereby enhancing B cell activation and antibody production targeting shared or adjacent epitopes on CVB3 [49,50]. Furthermore, the fusion of multiple epitopes may exert an adjuvant-like effect by activating pattern recognition receptors such as Toll-like receptors, thereby enhancing dendritic cell activation and, subsequently, promoting helper T cell support for B cell maturation [49,51]. However, this process requires direct validation through dendritic cell tracing experiments. Similarly, virus-like particles containing multiple enterovirus epitopes have been demonstrated to induce higher neutralizing antibody titers in comparison to monovalent formulations, suggesting that rCV-A3V synergistically enhances CVB3-specific immune responses through bystander activation or cytokine modulation [49,51,52]. More importantly, IgG antibodies against multiple EVA71, CVA16, and CVA6 epitope peptides were detected in the sera after the third immunization, and dominant epitopes were successfully identified, further confirming the immunogenicity of rCV-A3V and its precise epitope design. The strategy of integrating B cell, CTL, and Th epitopes reflects the limitation that a single epitope cannot fully cover the complete immune mechanism, necessitating the simultaneous activation of immune responses through multi-epitope combinations; this approach is consistent with the design concept of the HBoV1 vaccine [53].

The intranasal immunization strategy successfully activated broad and potent mucosal immune responses. Specific sIgA and IgG antibodies were detected in the nasal lavage fluid, feces, and milk, indicating that the vaccine-induced mucosal immunity covered the primary viral entry routes as well as the maternal transfer pathway, suggesting significant potential for anti-infection protection. Notably, the high levels of specific sIgA were detected in the milk of lactating mice immunized with rCV-A3V, indicating the potential of this intranasal vaccine in providing passive immunity to newborns through maternal antibodies, which, thereby, provides the key immunological basis for subsequent validation of its maternal transfer efficacy, although direct evidence from viral challenge experiments in neonatal suckling mice remains lacking. Furthermore, the presence of sIgA in the nasal lavage fluid and feces suggests lymphocyte homing to the mucosal tissues, although this mechanism warrants further confirmation through the detection of antigen-specific lymphocytes in mucosal tissues. Intranasal vaccines have established precedents as effective tools against various infectious diseases; for example, the oral live attenuated poliovirus vaccine has played a pivotal role in outbreak control [54]. The potent mucosal immunity observed in this study provides strong support for exploring non-invasive delivery methods such as nasal sprays or inhalable aerosols. Currently, several intranasal COVID-19 vaccines have entered clinical trials; for instance, an influenza virus vector-based nasal spray vaccine demonstrated cross-protection in hamsters for up to 9 months, further supporting the application prospects of mucosal immunization strategies [55].

rCV-A3V induced cellular immunity, as characterized by Th1 and CTL responses. Flow cytometry analysis revealed that the vaccine significantly promoted overall T cell proliferation, particularly of CD8^+^ T cells, exhibiting a Th1-biased profile. This observation aligns with the findings that intranasal administration of live attenuated influenza vaccines preferentially activates tissue-resident memory T cells (TRMs) and nasal-associated lymphoid tissue (NALT), thereby inducing stronger antigen-specific T cell proliferation compared to that by intramuscular injection. The observed reduction in the proportion of CD4^+^ T cells in this study may be attributed to multiple mechanisms. First, as an enterovirus, the CVB3 vector can target host immune regulatory factors through its encoded 3C protease (e.g., cleaving TFEB), thereby interfering with the survival and differentiation of CD4^+^ T cells [56]. Second, the marked expansion of CD8^+^ T cells during robust immune responses may have led to the relative reduction in the proportion of CD4^+^ T cells; concurrently, CD4^+^ T cells might be reduced due to activation-induced cell death or exhaustion [57]. Furthermore, mucosal immunization routes may preferentially activate local CD8^+^ T cell responses, with CD4^+^ T cells decreasing as they migrate to mucosal sites [47,58]. The significant elevation of IFN-γ indicates strong Th1 polarization, which is consistent with the ability of LAIV to upregulate Th1 cytokines in the NALT and spleen [59,60]. Notably, the current cellular immunity data represent only a foundational level; a more comprehensive evaluation of the quality of vaccine-induced cellular immunity warrants further in-depth functional analysis. Subsequent studies should therefore include direct assessment of the specific killing capacity of CD8^+^ T cells through in vivo CTL killing assays [61]. The identification of TRM subpopulations in the mucosal tissues using markers such as CD69, CD103, and CD49a [62,63], and the evaluation of the proportion of multifunctional T cells by detecting the capacity of antigen-specific T cells to co-express cytokines such as IFN-γ, TNF-α, and IL-2 via multi-parametric flow cytometry [64,65,66].

The intranasal live attenuated vaccine rCV-A3V successfully induced humoral, mucosal, and cellular immune responses in Balb/c mice, providing a foundation for the development of an HFMD vaccine with broad-spectrum protective potential and the possible capacity to interrupt viral transmission. However, this study has several limitations. First, the challenge protection data were not available. Limitations in suitable animal models have remained a persistent obstacle in HFMD vaccine research. Although the specific antibodies and T-cell responses observed in this study suggest potential protective efficacy, they provide only indirect evidence. The gold standard for evaluating vaccine protection remains validation through challenge experiments in susceptible animal models. Follow-up studies should focus on identifying suitable challenge models. Previous research has reported the successful evaluation of CVA16 vaccine efficacy using a neonatal Kunming mouse challenge model. Building on the findings of the present study, future work will focus on the following: (i) generating mouse-adapted strains of EV-A71, CVA16, and CVA6 to establish a stable and lethal challenge model in neonatal mice [67,68]. (ii) evaluating the protective efficacy of rCV-A3V against lethal viral challenge using an active immunization-challenge model; and (iii) validating the passive protective effect of maternal antibodies using a maternal immunization-neonatal challenge model. Second, live attenuated viral vectors carry an inherent risk of potential reversion to virulence. Although previous studies have verified the genetic stability of rCVB3 (mu) through serial in vitro passaging and RNA deep sequencing, and have assessed its attenuation characteristics through temperature-sensitivity testing and in vivo safety experiments, the long-term risk of virulence reversion cannot be completely excluded. Monitoring short-term changes in body weight alone is insufficient to fully evaluate this risk. Further attenuation of the vector should be considered in future studies. For example, introducing multiple point mutations into the 5′ untranslated region of CVB3 to disrupt the secondary structure of the internal ribosome entry site could reduce the likelihood of reversion to virulence [47,69,70]. The genetic stability of the rCV-A3V epitope cassette has yet to be evaluated. As exogenous sequences may destabilize the CVB3 vector and lead to sequence loss [71], subsequent studies should directly assess this stability through in vitro serial passaging.

Third, the epitopes incorporated into rCV-A3V were selected based on data from the human immune epitope database, and the robust immune responses elicited in Balb/c mice demonstrate the cross-species immunogenicity of these epitopes. However, substantial differences exist in antigen presentation between human and murine MHC molecules. Human HLA class I molecules can bind an exceptionally broad repertoire of antigenic peptides, whereas the peptide-binding repertoires of murine alleles, such as H-2Kb, are relatively restricted. This disparity may directly influence the observed immunogenicity. This suggests that the observed immunological effects may either underestimate or overestimate the vaccine’s potential efficacy in humans. In this study, a cross-species screening strategy of “human prediction + murine validation” was adopted; however, binding predictions were not specifically performed for mouse MHC (H-2) alleles. This approach was chosen because the ultimate goal of this research was to develop an HFMD vaccine for human use, and therefore, the primary criterion for epitope selection was high affinity for human HLA molecules. Although prediction tools for mouse H-2 molecules are available, the sequence similarity between murine MHC haplotypes and human HLA molecules is limited. Consequently, relying solely on mouse MHC predictions might exclude epitopes with potential efficacy in humans.

To address uncertainties arising from interspecies MHC differences, future studies could incorporate humanized HLA transgenic mouse models to verify epitope immunogenicity in a human-relevant context [72,73,74,75]. Fourth, the dosage regimen used in this study was determined based on the viral titers obtained after amplification and has not undergone systematic dose-ranging studies to establish the minimum effective dose or optimal immunization protocol. Future studies should therefore include multiple dose groups to compare the magnitude of immune responses and safety profiles induced by different doses. The dose-immunogenicity relationship should subsequently be validated in humanized HLA transgenic mice or non-human primates to provide a more reliable basis for dose selection in human studies.

Fifth, the immunizing doses differed between the vector control group and the vaccine group. The insertion of exogenous epitopes may have compromised the replication capacity of the recombinant virus in Vero cells, resulting in lower titers than those of the empty vector virus. In addition, recombinant viruses are more susceptible to activity loss and aggregation during the purification process [76,77]. To maintain a consistent immunization volume, mice in the two groups received different doses via intranasal administration. Although the dose administered to the rCV-A3V group was lower than that given to the rCVB3 (mu) group, rCV-A3V still induced stronger and broader immune responses. For example, after the second booster immunization, mice in the rCV-A3V group exhibited significantly higher levels of serum-specific IgG antibodies against EVA71, CVA16, CVA6, and CVB3, as well as higher levels of specific sIgA antibodies in nasal lavage fluid, compared to the rCVB3 (mu) group. These results suggest that the rCV-A3V vaccine possesses superior immunogenicity, potentially attributable to the introduction of exogenous epitopes that enhance both the breadth and magnitude of immune recognition and response. Notably, this effect was not diminished by the lower immunization dose. Therefore, the current experimental data may underestimate the true immunological potential of the vaccine. Previous studies have shown that even with reduced viral titers, engineered viral vectors can enhance immune responses through improved MHC-I/II presentation pathways [78,79]. However, the dose mismatch limits precise quantification of the immune contribution of the empty vector itself. Future studies should optimize virus purification processes to enable accurate evaluation of the specific immune efficacy of rCV-A3V under conditions with consistent immunization doses. The innovative aspect of this study lies in the combined strategy of multi-epitope design, a live attenuated viral vector, and intranasal immunization, resulting in a candidate vaccine capable of inducing both systemic and mucosal immunity at the primary site of infection. Our experimental data strongly support the potential of the rCV-A3V vaccine to protect against multiple HFMD pathogens, thereby laying a solid foundation for further evaluation of protective efficacy. This characteristic is particularly important in regions where EVA71, CVA16, and CVA6 co-circulate. Successfully addressing these challenges will provide further evidence supporting rCV-A3V as a safe and promising candidate HFMD vaccine.

## 5. Conclusions

To develop a multivalent vaccine against HFMD, we successfully constructed a multivalent epitope vaccine using rCVB3 (mu) as the viral vector. Fusion of antigenic epitopes derived from EV-A71, CVA16, and CVA6 with the attenuated vector, followed by intranasal administration, effectively induced robust mucosal, cellular, and humoral immune responses. Collectively, these findings demonstrate the broad immunogenicity of the rCV-A3V vaccine. In the preliminary safety evaluation, monitoring of mouse body weights revealed no significant evidence of acute toxicity. Combined with our laboratory’s previous research on the genetic stability, temperature sensitivity, and in vivo safety of CVB3 (mu) [48], this vaccine strategy appears to have a favorable safety profile and provides new insights for the development of multivalent, highly effective, and broad-spectrum enterovirus vaccines.

## Figures and Tables

**Table 1:** Candidate Linear B-Cell Epitopes of rCV-A3V, Candidate Cytotoxic T Lymphocyte (CTL) Epitopes of rCV-A3Vand Candidate T-helper (Th) Epitopes of rCV-A3V. The asterisk (*) denotes an allele, separating the locus name from the allele designation.

**Name**	**Sequence**		**Start Position**	**Toxin**	**Percent of Protein Sequence Matches at Identity 100%**		**Minimum Identity**	**Immunogenicity**	
EV-A71-1	VPPGAPKPDSRESLAW		721	Non-toxin	49.68% (844/1699)		31.25%	0.64952	
EV-A71-2	PTFGEHKQEKDLEYGA		774	Non-toxin	55.56% (944/1699)		25%	0.677406	
CVA16-1	WQTATNPSVFVKMTDP		736	Non-toxin	10.65% (181/1699)		37.5%	0.467135	
CVA16-2	YDGYPTFGEHLQANDL		770	Non-toxin	10.36% (176/1699)		31.25%	0.584572	
CVA6-1	PTFGEHKQATNLQYGQ		769	Non-toxin	21.95% (373/1699)		25%	0.850749	
CVA6-2	ASITTTDYEGGVPANP		851	Non-toxin	20.54% (349/1699)		25%	0.690303	
**Name**	**Sequence**		**Start Position**	**Allele**			**Toxin**	**Immunogenicity**	
EV-A71-1	SMINNIIIR		2030	HLA-A*31:01 HLA-A*68:01 HLA-A*11:01 HLA-A*03:01			Non-toxin	0.34575	
EV-A71-2	ISKFIDWLK		1136	HLA-A*11:01 HLA-A*68:01 HLA-A*30:01 HLA-A*31:01			Non-toxin	0.37874	
CVA16-1	REQGWIIPE		1478	HLA-B*40:01 HLA-B*35:01			Non-toxin	0.44332	
CVA16-2	EVTWENATF		2097	HLA-A*26:01			Non-toxin	0.3826	
CVA6-1	MINNIIIRA		2038	HLA-A*31:01 HLA-A*68:01			Non-toxin	0.38883	
CVA6-2	ATGIVTIWY		513	HLA-A*01:01 HLA-A*30:02			Non-toxin	0.4634	
**Name**	**Sequence**	**Start Position**	**Allele**	**Percentile Rank**	**Smm-ic50**	**IFN-γ Inducer**	**IL-4** **Inducer**	**IL-10** **Inducer**	**Immunogenicity**
EV-A71-1	PASAYQWFYDGYPTF	762	HLA-DRB3*01:01	0.38	116	Positive	Non-inducer	Non-inducer	0.781705
EV-A71-2	VRIYMRMKHVRAWIP	814	HLA-DRB1*11:01 HLA-DRB3*02:02	0.11	48	Positive	Non-inducer	Non-inducer	0.436475
CVA16-1	WDFGLQSSVTLVVPW	480	HLA-DRB1*04:01	0.77	51	Positive	Non-inducer	Non-inducer	0.060969
CVA16-2	TAVQVLPTAANTEAS	586	HLA-DRB1*08:02	0.32	295	Positive	Non-inducer	Non-inducer	0.436964
CVA6-1	RPILRTATVQGPSLD	1547	HLA-DRB1*08:02	0.58	409	Positive	Non-inducer	Non-inducer	0.442277

**Table 2:** Physicochemical Properties of the Candidate Vaccine rCV-A3V.

Immunogenicity	Toxicity	Allergenicity	Estimated Half-Life	Instability Index	Aliphatic Idex	Grand Average of Hydropathicity (GRAVY)
0.4635	Non-Toxin	Non-allergen	20 h (mammalian reticulocytes, in vitro) 30 min (yeast, in vivo) >10 h (Escherichia coli, in vivo)	36.80	64.59	−0.523
Number of amino acids: 292aa Molecular weight: 32.1414KDa Theoretical pI: 8.93						

## Data Availability

All primary data generated or analyzed during this study are included in this manuscript in the form of tables and figures. Further details are available from the corresponding author upon a reasonable request.

## Supplementary Materials

The following supporting information can be downloaded at: https://www.mdpi.com/article/10.3390/vaccines14040294/s1, Experimental Step 1: Virus Purification. Experimental Step 2: Western blot. Experimental Step 3: Immunofluorescence.
