# Supplementary material for: Construction of the Multi-Epitope HFMD Vaccine Based on an Attenuated CVB3 Vector and Evaluation of Immunological Responses in Mice
Source: Vaccines (Basel). 2026 Mar 26;14(4):294. doi: 10.3390/vaccines14040294 (PMC13119997; doi:10.3390/vaccines14040294)
Supplement: Supplementary file 1 [file vaccines-14-00294-s001.zip › Animal Ethics Review Form.pdf]

汕 头 大 学 医 学 院

汕头大学医学院伦理委员会批准函

批准号：SUMC-2021-17

汕头大学医学院伦理委员会受理了以下项目提出的关于生物医学伦理审查的申请。

项目名称：基于表位优化和重组病毒载体黏膜转运的多价手足口病肠道病毒黏膜疫苗的分子设计及免疫研究

项目负责人：李蕊

研究时间： 2022.1.1-2025.12.31

经汕头大学医学院伦理委员会讨论审查，该项目研究内容和过程遵循国际及国家颁布的有关生物医学研究的伦理要求，同意开展本项研究。

汕头大学医学院伦理委员会

汕头大学医学院伦理委员会

2021年 3 月 14 日
