# Supplementary material for: Construction of the Multi-Epitope HFMD Vaccine Based on an Attenuated CVB3 Vector and Evaluation of Immunological Responses in Mice
Source: Vaccines (Basel). 2026 Mar 26;14(4):294. doi: 10.3390/vaccines14040294 (PMC13119997; doi:10.3390/vaccines14040294)
Supplement: Supplementary file 1 [file vaccines-14-00294-s001.zip › Supplementary Materials.pdf]

Supplementary Materials

Virus Purification: Vero cells were infected with EVA71, CVA16, CVB3 (mu), and rCV-A3V at an MOI of 0.1, whereas RD cells were infected with CVA6 at an MOI of 0.1. The infections were performed in a total of 100 cell culture plates for each virus type. The cells were cultured at 37°C under 5% CO_2 for 72 h. After observing a complete cytopathic effect (CPE), the culture supernatants were harvested and pooled to obtain approximately 1 L of virus-containing supernatant for each virus type. Cellular debris was removed via centrifugation at 6,000 ×g for 30 min at 4°C. Viruses were precipitated from the clarified supernatant by adding 8% (w/v) polyethylene glycol 8000 and 0.3 M NaCl in sterile PBS, gently mixing, and then incubating overnight at 4°C. PEG precipitation is a gentle method for virus concentration that can be performed at 4°C, minimizing the loss of viral particle integrity and infectivity. Furthermore, PEG 8000 effectively precipitates enteroviruses. The precipitate was collected by centrifugation at 20,000 ×g for 30 min at 4°C in 250-mL centrifuge bottles. The resulting pellet was gently resuspended in 1 mL of 1X PBS. The resuspended virus solution was clarified via centrifugation at 13,400 ×g for 20 min, and the supernatant was collected. Density gradients were prepared by bottom-loading using a density gradient loading needle (with a syringe). The lowest concentration sucrose solution was first introduced to the bottom of the centrifuge tube, followed by the next higher concentration sucrose solution, which was slowly introduced from the bottom using the loading needle, allowing the higher-density solution to gently displace the lower-density solution. After preparing the density gradient, the virus sample was layered onto the gradient using a pipette. The tubes were balanced using a scale with an accuracy of 0.0001 g. Ultracentrifugation was then performed at 120,000 × g for 3.5 hours at 4°C under preset pressure (<20), temperature, and rotation speed conditions. Following centrifugation, the distinct virus band was carefully collected from the gradient via side puncture using a needle and syringe. The collected virus fraction was then dialyzed against 1X PBS at 4°C overnight using dialysis tubing, with two changes of 2-L dialysis buffer each. Finally, the purified virus was aliquoted into 1.5-mL microcentrifuge tubes and stored at -80°C for subsequent experiments.

Western Blot: A 12% SDS-PAGE gel was prepared using a commercial gel preparation kit (Beyotime, P0012AC). Electrophoresis was performed initially at 80 V for 30 min and then at 110

V for 1 h. Protein transfer was conducted using the wet transfer method. A PVDF membrane of appropriate size was activated by brief immersion in methanol for 30 s, followed by equilibration in a transfer buffer for 5 min. Transfer sponges and filter papers were also soaked in the transfer buffer. A transfer sandwich was assembled in the following order: sponge, filter paper, PVDF membrane, gel, filter paper, and sponge. The assembly was placed in a transfer tank filled with the buffer. Transfer was conducted at a constant current of 220 mA for 1 h. Following transfer, the membrane was blocked by incubation in 5% (w/v) skim milk prepared in TBST for 1 h at room temperature with gentle agitation. The membrane was then washed three times for 10 min each with TBST. Subsequently, the membrane was incubated overnight at 4°C with a 1:1000 dilution of the primary anti-Flag mouse monoclonal antibody (Beyotime, AF519) in the recommended diluent. The next day, the membrane was washed three times with TBST (10 min for each wash) and then incubated with a 1:1000 dilution of a HRP-conjugated goat anti-mouse IgG secondary antibody (Beyotime, A0216) for 1.5 to 2 h at room temperature. After incubation, the membrane was washed three times with TBST (10 min for each wash). Finally, chemiluminescence solution A and B (MedChemExpress, HY-K1005, USA) were mixed at a 1:1 ratio and evenly applied onto the membrane. The membrane was incubated with the chemiluminescent substrate for 1 minute and developed using a chemiluminescence imaging system. Exposure was performed in manual mode with exposure times ranging from 10 s, 20 s, 30 s, 40 s, 50 s, to 1 min. The optimal exposure image with moderate signal intensity and a clear background was selected for result presentation (as shown in Figure 2). The exposure time for β-Tubulin was 10 s, while that for rCV-A3V was 30 s.

Immunofluorescence: Vero cells were uniformly seeded in a 6-well plate and infected with rCV-A3V P1 at an MOI of 0.1 when the cell density reached near confluence. On observing 60–70% CPE, the culture medium was aspirated, and the cells were washed twice with PBS. Subsequently, the cells were fixed with 1 mL/well of 2–4% paraformaldehyde for 20 min at room temperature. After removing the fixative, the cells were washed thrice with PBS for 5 min each. Permeabilization was then performed using 0.5% Triton X-100 for 20 min at room temperature, followed by three additional 5-min PBS washes. Non-specific binding sites were blocked by incubation with 1% BSA for 1 h at room temperature. Following three 5-min washes with PBS,

the cells were incubated overnight at 4°C with a 1:1000 dilution of the primary anti-Flag mouse monoclonal antibody (Beyotime, AF519) prepared in the blocking buffer. The next day, the primary antibody solution was collected, and the cells were washed three times with PBS (5 min each time). A Cy3-conjugated secondary antibody (Beyotime, A0521) was then applied, and the plate was incubated for 1 h at room temperature in the dark. After three final 5-min PBS washes, the cell nuclei were counterstained with DAPI staining solution for 5 min on a shaking platform in the dark. Excess DAPI was removed by performing three additional 5-min PBS washes. Finally, the cells were visualized under a fluorescence microscope to detect an orange-red fluorescent signal.
